# Coronary Artery Calcium Scoring for Risk Reclassification and Prediction of Hard Cardiovascular Events in Asymptomatic Adults at Low-to-Intermediate Cardiovascular Risk: A Systematic Review

**DOI:** 10.7759/cureus.109433

**Published:** 2026-05-22

**Authors:** Ahmad Mohammad, Ahmad Irshad, Raja Faizan Aurangzeb, Salik Mahmood, Bhavna Singla, Shivam Singla, Fawad Haroon, Muhammad Junaid, Asad Talha

**Affiliations:** 1 Internal Medicine, Hurley Medical Center, Flint, USA; 2 Internal Medicine, Combined Military Hospital, Muzaffarabad, Muzaffarabad, PAK; 3 Internal Medicine, Fauji Foundation Hospital, Rawalpindi, PAK; 4 Cardiology, Aga Khan University Hospital, Karachi, PAK; 5 Internal Medicine, Erie County Medical Center Health Campus, Buffalo, USA; 6 Internal Medicine, TidalHealth Peninsula Regional, Salisbury, USA; 7 Accident and Emergency, Bahawal Victoria Hospital, Bahawalpur, PAK; 8 Internal Medicine, Jinnah Medical and Dental College, Karachi, PAK

**Keywords:** asymptomatic adults, cac score, cardiovascular risk stratification, coronary artery calcium, coronary heart disease, primary prevention, prospective cohort studies, risk reclassification

## Abstract

Coronary artery calcium (CAC) scoring has emerged as a valuable tool for cardiovascular risk assessment, yet its role in asymptomatic individuals at low-to-intermediate risk remains an area of ongoing investigation. This systematic review aimed to evaluate the prognostic value of CAC scoring for predicting hard cardiovascular events and its utility in risk reclassification beyond traditional risk models. A comprehensive literature search was conducted across PubMed/MEDLINE, Embase, and the Cochrane Library for studies published between 2000 and 2025. Prospective cohort studies assessing CAC in asymptomatic adults without established cardiovascular disease and reporting hard coronary outcomes were included. A total of eight studies met the inclusion criteria. Across these studies, CAC demonstrated a consistent and graded association with incident coronary heart disease events, independent of conventional risk factors. Higher CAC scores were associated with significantly increased risk, with several studies reporting markedly elevated risk at CAC ≥100, including hazard ratios approaching 4.6 in low-risk populations, while a CAC score of zero was consistently linked to very low short- to intermediate-term event rates. Importantly, CAC improved risk stratification, particularly among individuals initially classified as intermediate risk, with studies reporting substantial risk reclassification and net reclassification improvement values of approximately 0.25. Evidence also suggested that CAC is more strongly predictive of coronary events than stroke, supporting its role as a coronary-specific risk marker. Although the review included only eight studies and demonstrated methodological heterogeneity in CAC categorization, comparator models, and outcome definitions, findings were directionally consistent across diverse populations and study designs. These results indicate that CAC scoring provides incremental prognostic value and may serve as a clinically useful tool to refine risk assessment and guide individualized preventive strategies in asymptomatic adults at low-to-intermediate cardiovascular risk.

## Introduction and background

Cardiovascular disease remains the leading cause of morbidity and mortality worldwide, with coronary heart disease accounting for a substantial proportion of this burden. A central challenge in contemporary preventive cardiology is the accurate identification of asymptomatic individuals who are at increased risk of future events but may not be adequately captured by traditional risk assessment tools [1]. Widely used models, such as the Framingham Risk Score and pooled cohort equations, rely on demographic and clinical variables to estimate risk; however, these approaches may both underestimate and overestimate risk in certain populations. This limitation is particularly evident among individuals classified as low to intermediate risk, in whom clinical decision-making regarding preventive therapies often remains uncertain [2,3].

Coronary artery calcium (CAC) scoring, obtained through non-contrast cardiac computed tomography, has emerged as a direct measure of subclinical atherosclerotic burden. Unlike traditional risk factors that infer risk indirectly, CAC reflects the cumulative effect of atherosclerosis over time and has demonstrated a strong association with incident coronary events in diverse populations [4,5]. Numerous prospective cohort studies have shown that higher calcium scores are associated with a graded increase in the risk of myocardial infarction and coronary death, independent of conventional risk factors. In addition, the absence of coronary calcification has been associated with very low short- to intermediate-term event rates, supporting its potential role in identifying individuals who may safely defer intensive preventive interventions [4].

Beyond its role as a prognostic marker, CAC has gained attention for its ability to refine cardiovascular risk stratification. The concept of risk reclassification, whereby individuals are reassigned to more appropriate risk categories after incorporation of additional biomarkers, is of particular relevance in asymptomatic populations [6]. Evidence suggests that CAC scoring can meaningfully reclassify individuals initially categorized as intermediate risk into higher or lower risk strata, thereby influencing management decisions such as initiation of statin therapy. However, variability exists across studies in terms of population characteristics, outcome definitions, and analytic approaches, and the extent to which CAC consistently improves risk classification and prediction of hard cardiovascular events in low- to intermediate-risk individuals remains an area requiring further synthesis [7,8].

The objective of this systematic review is to evaluate the prognostic value of CAC scoring for the prediction of hard cardiovascular events and its role in risk reclassification among asymptomatic adults at low to intermediate cardiovascular risk, based on evidence from prospective cohort studies.

## Review

Materials and methods

Study Design and Reporting Standards

This systematic review was conducted in accordance with the Preferred Reporting Items for Systematic Reviews and Meta-Analyses (PRISMA) guidelines to ensure methodological transparency and reproducibility [9]. A structured and predefined methodology was followed, encompassing study identification, screening, eligibility assessment, data extraction, quality appraisal, and synthesis. The review was designed to evaluate the prognostic value of CAC scoring and its role in risk reclassification among asymptomatic adults in primary prevention settings.

Eligibility Criteria

Eligibility criteria were defined using the Population, Intervention, Comparator, Outcomes, and Study Design (PICOS) framework [10]. Studies were included if they evaluated asymptomatic adults without established cardiovascular disease, primarily within low-to-intermediate cardiovascular risk groups, and assessed CAC scoring obtained via computed tomography. Eligible studies were required to compare CAC with traditional cardiovascular risk models, such as Framingham-based or multivariable-adjusted risk scores, and report hard cardiovascular outcomes, including myocardial infarction, coronary heart disease death, or composite major coronary events. Studies reporting measures of risk reclassification, such as net reclassification improvement, were also included. Only prospective cohort studies were considered for inclusion to ensure methodological rigor and temporal validity. Studies were excluded if they involved symptomatic populations, established cardiovascular disease, non-coronary outcomes alone (such as isolated stroke or all-cause mortality), or were non-original research articles, including reviews, editorials, or case reports. To ensure relevance to contemporary clinical practice, studies published from approximately 2000 onward were included.

Search Strategy

A comprehensive literature search was performed across multiple major electronic databases, including PubMed/MEDLINE, Embase, and the Cochrane Library, to ensure broad coverage of relevant studies. The search encompassed studies published from January 2000 to December 2025, reflecting the era of contemporary CAC assessment and prospective cohort research. The search strategy combined Medical Subject Headings (MeSH) and free-text keywords to optimize sensitivity and specificity. Key search terms included “coronary artery calcium,” “coronary calcium score,” “coronary artery calcification,” “cardiovascular diseases,” “coronary heart disease,” “risk assessment,” “risk stratification,” “risk prediction,” “reclassification,” “asymptomatic,” and “primary prevention.” Boolean operators were applied systematically, using combinations such as (“coronary artery calcium” OR “CAC score” OR “coronary calcification”) AND (“cardiovascular events” OR “coronary heart disease” OR “myocardial infarction”) AND (“risk assessment” OR “risk stratification” OR “risk prediction” OR “reclassification”) AND (“asymptomatic” OR “primary prevention”). The search was limited to studies published in English. In addition, the reference lists of relevant articles and key reviews were manually screened to identify any additional eligible studies not captured through the database search.

Study Selection

Study selection was conducted in a two-stage process. Initially, titles and abstracts of retrieved records were screened for relevance based on predefined eligibility criteria. Subsequently, full-text articles of potentially eligible studies were assessed in detail to confirm inclusion. Duplicate records were removed prior to screening. Particular attention was given to identifying overlapping cohorts, especially within large longitudinal studies, and in such cases, studies were selected based on their methodological strength, relevance to the research question, and uniqueness of the analysis to avoid redundancy.

Data Extraction

Data extraction was performed using a standardized and structured approach to ensure consistency across included studies. Extracted variables included study characteristics (author, year, cohort, and country), sample size, participant demographics, risk profile of the population, CAC measurement methods and categorization, comparator risk models, duration of follow-up, primary and secondary outcomes, and key findings related to prognostic value and risk reclassification. Where necessary, CAC categories and outcome definitions were conceptually harmonized to facilitate comparison across studies while preserving the integrity of the original analyses.

Quality Assessment

The methodological quality of the included studies was assessed using the Newcastle-Ottawa Scale [11], which evaluates observational cohort studies across three domains: selection of participants, comparability of cohorts, and outcome assessment. Each study was assigned a score out of a maximum of nine stars. Studies scoring between 8 and 9 were considered high quality, while those scoring between 6 and 7 were considered moderate quality. This assessment was used to inform the interpretation of findings and to contextualize the strength of evidence.

Data Synthesis

Due to methodological heterogeneity across studies, including variations in CAC categorization, comparator models, and outcome definitions, a quantitative meta-analysis was not performed. Instead, a qualitative narrative synthesis was undertaken. Studies were analyzed thematically, focusing on three key domains: prediction of hard coronary events, risk reclassification beyond traditional models, and prognostic implications of CAC categories, including absent and minimal CAC. Patterns of association, dose-response relationships, and consistency of findings across diverse populations were systematically evaluated. Particular emphasis was placed on the incremental value of CAC in refining cardiovascular risk assessment in asymptomatic individuals at low-to-intermediate risk.

Handling of Heterogeneity

Given the variability in study designs and reporting methods, heterogeneity was addressed through conceptual rather than statistical approaches. Differences in CAC thresholds, outcome definitions, and follow-up durations were acknowledged and incorporated into the interpretation of findings. Rather than limiting synthesis, this variability allowed for assessment of the consistency of CAC’s prognostic value across diverse populations and methodological contexts, supporting a more comprehensive and generalizable understanding of its clinical utility.

Results

Study Selection Process

**Figure 1:**
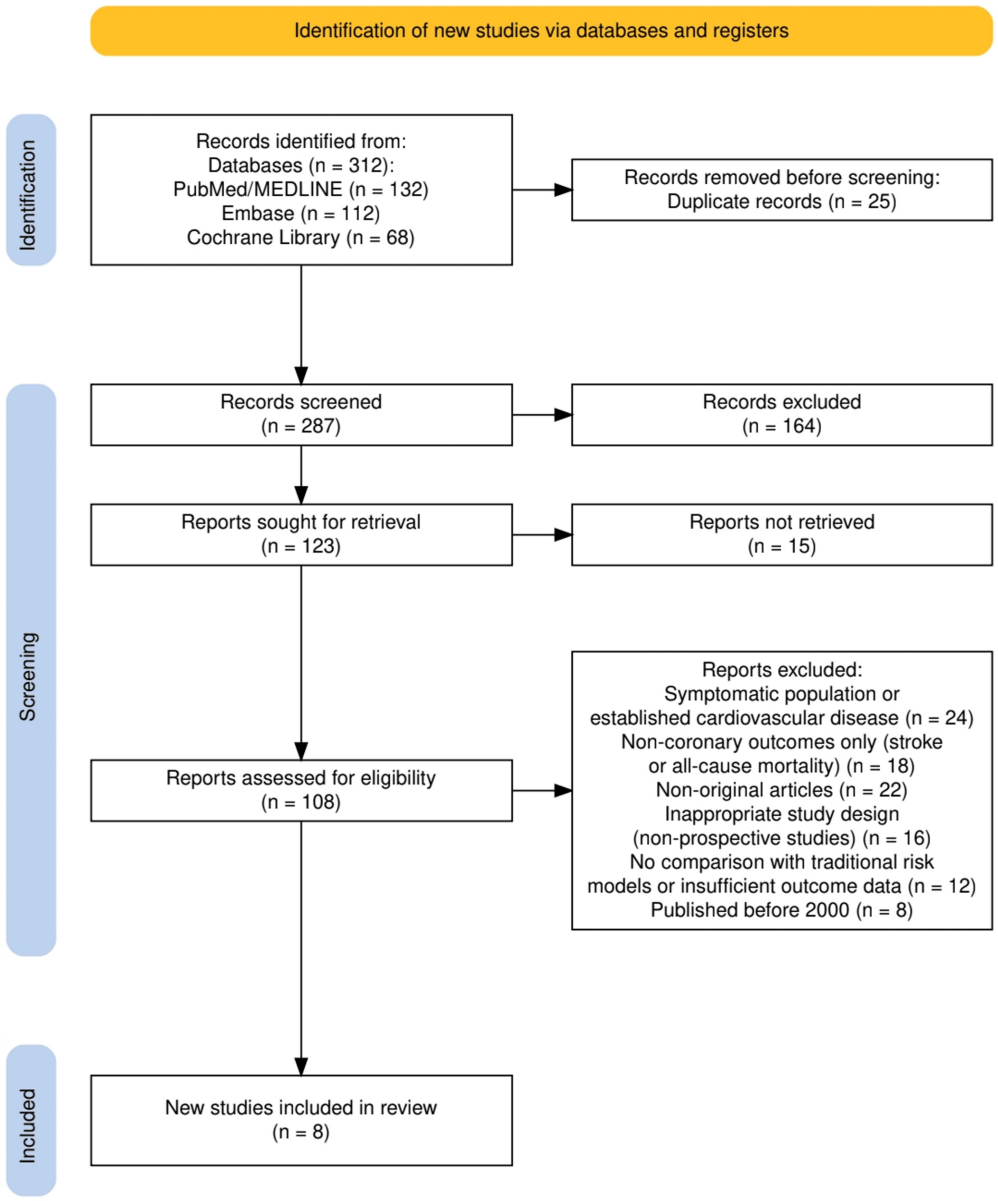
PRISMA flow diagram of study selection process for the systematic review. PRISMA: Preferred Reporting Items for Systematic Reviews and Meta-Analyses

A total of 312 records were identified through database searching, with 132 from PubMed/MEDLINE, 112 from Embase, and 68 from the Cochrane Library. After removal of 25 duplicate records, 287 studies underwent title and abstract screening, of which 164 were excluded based on irrelevance. Of the remaining 123 reports sought for retrieval, 15 were not accessible, leaving 108 full-text articles assessed for eligibility. Following application of predefined inclusion and exclusion criteria, 100 studies were excluded for reasons including symptomatic populations, non-coronary outcomes, non-original study designs, inappropriate methodology, insufficient data, or publication prior to 2000. Ultimately, eight studies met all eligibility criteria and were included in the final qualitative synthesis, as illustrated in Figure 1.

Characteristics of the Selected Studies

A total of eight prospective cohort studies were included in the final analysis, encompassing diverse populations across multiple geographic regions, including the United States and Europe. Sample sizes ranged from approximately 1,300 to over 7,000 participants, with cohorts primarily consisting of asymptomatic adults without established cardiovascular disease and predominantly within low-to-intermediate risk categories. The mean age varied across studies, reflecting inclusion of both younger and elderly populations, thereby enhancing generalizability. CAC was assessed using computed tomography and categorized using varying thresholds, most commonly including ranges such as 0, 1-99, 100-399, and ≥400. Comparator models typically involved traditional cardiovascular risk factors or Framingham-based risk scores. Follow-up durations ranged from approximately three to over 12 years, allowing for assessment of incident hard coronary outcomes, including myocardial infarction and coronary heart disease death. Despite methodological differences in CAC categorization and outcome definitions, all studies consistently evaluated the incremental prognostic value of CAC and its role in risk stratification, as summarized in Table 1.

**Table 1:** Characteristics of the included studies evaluating CAC for cardiovascular risk prediction and reclassification. CAC: coronary artery calcium; ASCVD: atherosclerotic cardiovascular disease; CHD: coronary heart disease; ACS: acute coronary syndrome; MI: myocardial infarction; CVD: cardiovascular disease; HR: hazard ratio; AUC: area under the curve; NRI: net reclassification improvement; MESA: Multi-Ethnic Study of Atherosclerosis; DHS: Dallas Heart Study; PACC: Prospective Army Coronary Calcium Project

Study (Year)	Cohort/Country	Sample Size	Population (Risk Profile)	Mean Age	CAC Categories	Comparator Model	Follow-Up	Outcomes (Hard Events)	Key Findings
Mehta et al. (2020) [12]	MESA + DHS/USA	7,042	Asymptomatic, multiethnic; primary prevention population (mixed risk, includes low-intermediate)	57 years	0, 1-99, ≥100	Traditional risk factors	12.3 years	ASCVD events (CHD + stroke), CHD, stroke	CAC strongly predicted CHD across all sex and race groups; ≥100 associated with higher ASCVD risk; improved discrimination and reclassification for CHD but not stroke
Taylor et al. (2005) [13]	PACC/USA	2,000	Asymptomatic, predominantly low-risk; young adults (40-50 yrs)	43 years	Presence vs absence; tertiles of CAC severity	Framingham Risk Score	3.0 years	Primary: CHD events (ACS, sudden cardiac death)	CAC independently predicted premature CHD; ~12-fold increased risk; incremental value beyond Framingham; dose-response across CAC tertiles
Elias-Smale et al. (2010) [14]	Rotterdam Study/Netherlands	2,028	Asymptomatic, elderly general population; low-intermediate risk stratified	69.6 years	Continuous CAC; derived cutoffs (<50, >615)	Framingham Risk Score (refitted)	9.2 years	Primary: Hard CHD events	CAC significantly improved risk classification, especially in intermediate-risk individuals; ~52% reclassified; cutoffs <50 (low risk) and >615 (high risk)
Joshi et al. (2016) [15]	MESA/USA	1,391 (low lifetime risk subgroup)	Asymptomatic adults with low lifetime cardiovascular risk	Not specified	0, >0, >100	Multivariable-adjusted risk factors	10.4 years	Primary: CHD events	CAC was the strongest predictor of CHD; CAC >100 independently predicted events (HR ~4.6); clear gradient of risk (CAC 0 vs >0 vs >100)
Budoff et al. (2009) [16]	MESA/USA	3,923 (CAC 0-10 subgroup)	Asymptomatic adults; low-intermediate risk subset (CAC 0 or minimal)	58 years	0 vs 1-10	Multivariable-adjusted risk factors	4.1 years	Primary: Hard CHD events (MI, CHD death); Secondary: All CHD events	Minimal CAC (1-10) associated with ~3-fold higher risk vs CAC = 0; CAC = 0 associated with very low event rates (“power of zero”)
Budoff et al. (2009) [17]	MESA/USA	6,814	Asymptomatic, multiethnic; primary prevention (mixed risk including low-intermediate)	Not specified	0, 1-100, 101-400, >400 vs percentile-based CAC	Multivariable-adjusted risk factors	3.75 years	Primary: CHD events	Absolute CAC scores predicted CHD events better than age/sex/race percentiles; improved discrimination (higher AUC); risk increased consistently across absolute CAC categories
Polonsky et al. (2010) [18]	MESA/USA	5,878	Asymptomatic adults without CVD; predominantly low-intermediate risk (diabetics excluded)	Not specified (~early 60s typical MESA)	Continuous CAC + categories	Traditional risk model (Framingham-like variables) vs + CAC	5.8 years	Primary: CHD events (MI, CHD death, cardiac arrest)	Addition of CAC significantly improved risk classification (NRI = 0.25); more individuals were correctly reclassified into high- and low-risk categories
Detrano et al. (2008) [19]	MESA/USA (multiethnic)	6,722	Asymptomatic adults without CVD; primary prevention (low-intermediate risk mix)	Not specified (~early 60s typical MESA)	0, 1-100, 101-300, >300	Traditional risk factors	3.8 years	Primary: Major CHD events (MI, CHD death); Secondary: Any CHD events	CAC strongly predicted CHD across all ethnic groups; high CAC (≥101) associated with markedly increased risk (HR ~7-10); improved discrimination beyond traditional risk factors

Quality Assessment

The methodological quality of the included studies was assessed using the Newcastle-Ottawa Scale, with most studies demonstrating high quality. Overall scores ranged from 7 to 9, reflecting strong performance in participant selection, appropriate adjustment for confounding variables, and reliable outcome assessment. The majority of studies achieved maximum scores in the selection and comparability domains, supported by well-defined asymptomatic cohorts and multivariable-adjusted analyses. Slightly lower scores in a few studies were primarily due to shorter follow-up durations or lower event rates. Despite minor variations, the overall quality of evidence was robust, supporting the validity of the findings presented, as summarized in Table 2.

**Table 2:** Quality assessment of included studies using the Newcastle-Ottawa Scale (NOS). Selection: Assessment of cohort selection and representativeness (maximum four stars); Comparability: Adjustment for confounding variables (maximum two stars); Outcome: Assessment of outcome measurement and follow-up adequacy (maximum three stars)

Study (Year)	Selection (4)	Comparability (2)	Outcome (3)	Total (9)	Quality
Mehta et al. (2020) [12]	★★★★	★★	★★★	9	High
Taylor et al. (2005) [13]	★★★	★★	★★	7	Moderate-High
Elias-Smale et al. (2010) [14]	★★★★	★★	★★★	9	High
Joshi et al. (2016) [15]	★★★★	★★	★★★	9	High
Budoff et al. (2009) [16]	★★★★	★★	★★	8	High
Budoff et al. (2009) [17]	★★★★	★★	★★	8	High
Polonsky et al. (2010) [18]	★★★★	★★	★★★	9	High
Detrano et al. (2008) [19]	★★★★	★★	★★★	9	High

Discussion

Principal Findings

This systematic review demonstrates that, among asymptomatic adults at low-to-intermediate cardiovascular risk, CAC scoring consistently provides incremental prognostic value beyond traditional risk assessment. Across multiple prospective cohorts, CAC showed a robust association with hard coronary heart disease events, with the strength of evidence most pronounced for coronary outcomes rather than broader vascular endpoints. Studies such as Detrano et al. [19] and Mehta et al. [12] confirm a clear dose-response relationship between increasing CAC burden and incident events, while Joshi et al. [15] highlight its relevance even in individuals classified as low risk by conventional criteria. Importantly, Polonsky et al. [18] and Elias-Smale et al. [14] demonstrated that CAC meaningfully improves risk classification, particularly in intermediate-risk populations. Collectively, these findings suggest that CAC should not be regarded merely as an associative biomarker, but rather as a clinically useful tool that refines decision-making in patients whose baseline risk remains uncertain.

Biological and Clinical Rationale

The added value of CAC over traditional risk models can be understood by considering the fundamental difference in what each approach measures. Conventional risk scores estimate probability based on demographic and clinical risk factors, which may incompletely capture the underlying burden of atherosclerosis. In contrast, CAC directly quantifies subclinical coronary plaque, reflecting the cumulative exposure to atherogenic processes over time. This distinction explains why CAC improves risk discrimination [20] and, in some contexts, calibration, as shown in studies such as Detrano et al. [19] and Budoff et al. [16], where the inclusion of CAC enhanced model performance beyond traditional variables. Furthermore, because CAC identifies established disease rather than inferred risk, it is particularly valuable in individuals whose treatment decisions are uncertain based on conventional assessment alone. In this sense, CAC can be conceptualized as a bridge between epidemiologic risk estimation and individualized disease detection, linking population-based prediction with direct evidence of coronary pathology [21].

Risk Reclassification as a Clinical Advantage

A central finding of this review is that the most clinically meaningful contribution of CAC lies in its capacity to improve risk reclassification rather than simply augment prediction. Traditional risk models often leave a substantial proportion of asymptomatic individuals in intermediate or indeterminate categories, where management decisions are less clear. Evidence from Polonsky et al. [18] demonstrated that the addition of CAC leads to significant net reclassification improvement, while Elias-Smale et al. [14] reported that approximately half of intermediate-risk individuals were reassigned to more appropriate risk categories. This reclassification operates bidirectionally, identifying individuals with subclinical disease who may benefit from intensified preventive strategies, while also recognizing those with minimal or absent calcification who may be at sufficiently low short-term risk to avoid unnecessary treatment. Similarly, Joshi et al. [15] showed that CAC can uncover higher-risk individuals even within populations initially classified as low risk. These findings suggest that CAC should not be viewed merely as a stronger predictive marker, but rather as a risk adjudication tool that resolves uncertainty and aligns estimated risk more closely with underlying disease burden [22].

Prognostic Significance of CAC = 0 and Minimal CAC

One of the most clinically informative observations across the included studies is the prognostic value of a CAC score of zero, alongside the recognition that minimal CAC is not equivalent to absent risk. Evidence from Budoff et al. [16] demonstrated that individuals with CAC = 0 have very low short- to intermediate-term rates of hard coronary events, supporting its role in identifying patients who may safely defer intensive preventive interventions. However, even minimal CAC (scores 1-10) was associated with a significantly higher risk compared with zero CAC, indicating that early detectable calcification carries meaningful prognostic information. Similar gradients in risk are observed in broader cohorts such as Joshi et al. [15], where increasing CAC burden correlates with higher event rates even within low-risk populations. These findings underscore that CAC should be interpreted as a continuum rather than a binary measure. The clinical utility of CAC lies not only in detecting substantial atherosclerotic burden, but also in distinguishing true absence of calcified disease from the earliest measurable stages of plaque development, thereby refining both reassurance and early intervention strategies [23].

Coronary Versus Cerebrovascular Risk Prediction

An important and often underemphasized finding is that the prognostic strength of CAC is more consistent for coronary events than for stroke. Data from Mehta et al. [12] showed that while CAC robustly predicts coronary heart disease outcomes across sex and racial groups, its predictive performance for stroke is comparatively limited. This distinction is biologically plausible, as CAC directly reflects coronary atherosclerotic burden, whereas stroke arises from a more heterogeneous set of mechanisms, including embolic, small vessel, and non-atherosclerotic etiologies. Findings from Detrano et al. [19] further reinforced the strong and consistent relationship between CAC and coronary events across diverse populations. Taken together, these observations suggest that CAC may be more appropriately conceptualized as a marker of coronary risk first, with broader but less uniform applicability to overall atherosclerotic cardiovascular disease (ASCVD). In this context, CAC should be interpreted not as a universal vascular risk marker, but as a coronary-specific indicator with secondary extrapolation to non-coronary outcomes [24].

Heterogeneity and Consistency Across Studies

The included studies demonstrate notable methodological heterogeneity, reflecting differences in CAC categorization, comparator risk models, follow-up durations, and definitions of hard coronary outcomes. For instance, CAC thresholds varied from binary comparisons to multi-category stratifications, while comparator models ranged from traditional Framingham-based approaches to multivariable-adjusted frameworks. Follow-up periods also differed substantially, from approximately three years in Taylor et al. [13] to over a decade in studies such as Joshi et al. [15] and Mehta et al. [12], potentially influencing event rates and observed associations. Additionally, several analyses were derived from large cohorts such as the Multi-Ethnic Study of Atherosclerosis (MESA), albeit focusing on distinct subpopulations, including low-risk individuals or those with minimal CAC. Despite this variability, a consistent pattern emerges across studies, with CAC demonstrating a graded and independent association with coronary events and improving risk stratification beyond conventional factors. Thus, while heterogeneity limited direct quantitative pooling, the literature exhibits methodological heterogeneity but directional coherence, strengthening the overall validity and generalizability of the findings [25].

Contribution to the Existing Literature

This review extends the existing body of evidence by providing a focused synthesis of the prognostic and clinical utility of CAC in asymptomatic adults at low-to-intermediate cardiovascular risk, with particular emphasis on hard coronary outcomes and risk reclassification. While prior studies, including those by Detrano et al. [19] and Polonsky et al. [18], have established the predictive value of CAC, the present analysis integrates these findings within a clinically relevant framework centered on populations in whom treatment decisions are most uncertain. By incorporating evidence from diverse cohorts such as Elias-Smale et al. [14], Joshi et al. [15], and Budoff et al. [17], this review highlights not only the predictive strength of CAC but also its role in refining risk categorization and guiding individualized prevention strategies. Furthermore, by distinguishing between coronary-specific prediction and broader vascular outcomes, this work provides a more nuanced interpretation of CAC’s applicability. Importantly, this review suggests that the true strength of CAC in asymptomatic primary prevention populations lies less in confirming overt high risk and more in resolving uncertainty among individuals not clearly classified as high or low risk by conventional assessment.

Limitations

Several limitations of the present synthesis should be acknowledged. First, the evidence base is predominantly derived from observational cohort studies, which, despite robust design and adjustment for confounders, remain subject to residual confounding and cannot establish causality. Second, a substantial proportion of the included analyses originates from the MESA, raising the possibility of cohort overlap, although distinct subpopulations and analytical approaches were considered to mitigate redundancy. Third, variability in CAC categorization, comparator risk models, and definitions of hard cardiovascular outcomes introduces heterogeneity that limits direct quantitative comparison across studies. Additionally, formal measures of risk reclassification, such as net reclassification improvement, were not consistently reported across all studies. Some investigations also prioritized coronary heart disease outcomes, whereas others included broader ASCVD or mortality endpoints, further contributing to variability. Finally, although this review focuses on low-to-intermediate risk populations, relatively fewer studies were exclusively designed to evaluate truly low-risk individuals, which may affect the generalizability of conclusions in this subgroup.

Clinical Implications and Future Directions

The findings of this review have important implications for clinical practice and future research. CAC scoring appears to be most valuable in asymptomatic individuals with uncertain treatment thresholds, particularly those classified as low to intermediate risk by traditional models, where it can refine risk assessment and support more individualized decision-making. The use of absolute CAC categories, as supported by studies such as Budoff et al. [17], offers a practical and clinically interpretable approach compared with percentile-based methods. Moving forward, further research is needed to standardize outcome definitions and CAC categorization to enhance comparability across studies. Dedicated analyses focusing specifically on low-risk populations would help clarify the role of CAC in early prevention strategies. Additionally, direct comparisons between CAC-guided assessment and contemporary risk prediction tools are warranted. Importantly, future studies should evaluate how CAC-based risk reclassification translates into changes in clinical management and, ultimately, whether such strategies improve long-term cardiovascular outcomes.

## Conclusions

In asymptomatic adults at low-to-intermediate cardiovascular risk, CAC scoring provides consistent and clinically meaningful incremental value beyond traditional risk assessment for the prediction of hard coronary events and refinement of risk classification. The evidence indicates that CAC is most impactful not as a standalone predictor of obvious high risk, but as a tool that resolves uncertainty by identifying individuals who may benefit from either intensification or de-escalation of preventive strategies. The prognostic significance of both absent and increasing CAC underscores its role as a continuum-based marker of subclinical atherosclerosis. Taken together, these findings support the integration of CAC into risk assessment frameworks for selected asymptomatic individuals, where it can enhance precision in primary prevention and guide more individualized clinical decision-making.
